# Entrapment neuropathy results in different microRNA expression patterns from denervation injury in rats

**DOI:** 10.1186/1471-2474-11-181

**Published:** 2010-08-12

**Authors:** Cheng-Shyuan Rau, Jonathan Chris Jeng, Seng-Feng Jeng, Tsu-Hsiang Lu, Yi-Chun Chen, Po-Chou Liliang, Chia-Jung Wu, Chia-Jung Lin, Ching-Hua Hsieh

**Affiliations:** 1 Department of Neurosurgery, Chang Gung Memorial Hospital - Kaohsiung Medical Center, Chang Gung University College of Medicine, Taiwan; 2 Business BA at University of Texas at Dallas, 800 W Campbell Road, Richardson, TX 75080, USA; 3 Department of Plastic and Reconstructive Surgery, Chang Gung Memorial Hospital - Kaohsiung Medical Center, Chang Gung University College of Medicine, Taiwan; 4 Department of Neurosurgery, E-Da Hospital, I-Shou University, Kaohsiung, Taiwan

## Abstract

**Background:**

To compare the microRNA (miRNA) expression profiles in neurons and innervated muscles after sciatic nerve entrapment using a non-constrictive silastic tube, subsequent surgical decompression, and denervation injury.

**Methods:**

The experimental L4-L6 spinal segments, dorsal root ganglia (DRGs), and soleus muscles from each experimental group (sham control, denervation, entrapment, and decompression) were analyzed using an Agilent rat miRNA array to detect dysregulated miRNAs. In addition, muscle-specific miRNAs (miR-1, -133a, and -206) and selectively upregulated miRNAs were subsequently quantified using real-time reverse transcription-polymerase chain reaction (real-time RT-PCR).

**Results:**

In the soleus muscles, 37 of the 47 miRNAs (13.4% of the 350 unique miRNAs tested) that were significantly downregulated after 6 months of entrapment neuropathy were also among the 40 miRNAs (11.4% of the 350 unique miRNAs tested) that were downregulated after 3 months of decompression. No miRNA was upregulated in both groups. In contrast, only 3 miRNAs were upregulated and 3 miRNAs were downregulated in the denervated muscle after 6 months. In the DRGs, 6 miRNAs in the entrapment group (miR-9, miR-320, miR-324-3p, miR-672, miR-466b, and miR-144) and 3 miRNAs in the decompression group (miR-9, miR-320, and miR-324-3p) were significantly downregulated. No miRNA was upregulated in both groups. We detected 1 downregulated miRNA (miR-144) and 1 upregulated miRNA (miR-21) after sciatic nerve denervation. We were able to separate the muscle or DRG samples into denervation or entrapment neuropathy by performing unsupervised hierarchal clustering analysis. Regarding the muscle-specific miRNAs, real-time RT-PCR analysis revealed an ~50% decrease in miR-1 and miR-133a expression levels at 3 and 6 months after entrapment, whereas miR-1 and miR-133a levels were unchanged and were decreased after decompression at 1 and 3 months. In contrast, there were no statistical differences in the expression of miR-206 during nerve entrapment and after decompression. The expression of muscle-specific miRNAs in entrapment neuropathy is different from our previous observations in sciatic nerve denervation injury.

**Conclusions:**

This study revealed the different involvement of miRNAs in neurons and innervated muscles after entrapment neuropathy and denervation injury, and implied that epigenetic regulation is different in these two conditions.

## Background

Chronic nerve compression affects millions of individuals and results in pain and loss of function. Dependent on the amount and duration of compression imposed on the nerve, the pathological changes associated with chronic nerve compression range from the breakdown of the blood-nerve barrier in the early stages, to subperineurial edema, fibrosis, demyelination, and eventually Wallerian degeneration, which can be associated with loss of two-point discrimination and muscle atrophy [1,2]. Surgical decompression of the nerve is warranted if the symptoms are refractory to conservative treatments; however, the reversal of motor weakness is usually limited and unpredictable [1]. Generally, denervation leads to significant changes in the innervated muscle, e.g., muscle atrophy. In contrast, reinnervation helps to reverse the change or to prevent further deterioration of the denervated muscle [3]. After nerve injury, the mismatch of the motor and sensory fibers of the mixed nerve in nerve microanastomosis, the existence of a long nerve defect, and a long distance from the injured area to the innervated muscle are considered to be the main factors leading to a worse functional outcome [4]. In entrapment neuropathy, considering that there are no aforementioned circumstances, it is unknown why a successful surgical decompression does not result in a predictable and satisfactory outcome; therefore, a better understanding of the mechanisms of induction and mediation of these conditioning responses is necessary.

MicroRNAs (miRNAs) are emerging as key modulators of post-transcriptional gene regulation in a variety of tissues, including the nervous system [5]. miRNAs are a novel regulatory class of non-coding, single-stranded RNAs of approximately 22 nucleotides that are implicated in a wide range of diverse genetic regulatory mechanisms [6,7]. Basic and clinical studies suggested that miRNAs are important regulators in normal physiological processes and diseases [8-11]. Many miRNAs are expressed in a tissue-specific manner. In neurons, miRNAs are expressed at all stages of development, and miRNA-dependent posttranscriptional gene regulation plays a pivotal role at all stages of neural development, including neural differentiation, morphogenesis, and plasticity [12]. Recent results also point to a role for miRNAs in axonal biology [13] as well as in the control of synaptic function and plasticity [5]. In addition, there is increasing evidence for the involvement of microRNAs in myopathies [14-16]. A number of microRNAs, including muscle-specific and non-muscle-specific miRNAs, have been characterized as regulators of skeletal muscle development and diseases [17-20] as well as of skeletal muscle remodeling [21]. Three muscle-specific miRNAs (miR-1, miR-133, and miR-206), with multiple key roles in the control of muscle growth and differentiation, have been the focus of intense research. We previously demonstrated that the expression of miR-1 and miR-133 in the soleus muscle of rats increased by ~2-fold at 4 months after sciatic nerve denervation and after reinnervation with microanastomosis [22]; however, the expression of miR-206 was significantly increased by 3-fold at 1 month later and lasted for at least 4 months after reinnervation, but not after denervation [22]. Moreover, the expression of miR-206 may play a role in determining fiber type after peripheral nerve regeneration via the downregulation of the Mef2 transcript [22]. It was suggested that the increased expression of miR-206 in newly formed myotubes may reflect active regeneration and efficient maturation of skeletal muscle fibers [23]. In a mouse model of the neurodegenerative disease amyotrophic lateral sclerosis (ALS), the role of miR-206 in the reinnervation of the neuromuscular junction after injury and in improving survival was also defined [24].

Given the importance of miRNAs, we were interested in the expression profile of miRNAs involved in entrapment neuropathy. We addressed this issue using a microarray-based screening approach in the neurons and innervated muscles involved in this condition, and compared the data with the expression patterns observed in denervation injury and after decompression surgery. In addition, the expression profiles of the upregulated neuron- and muscle-specific miRNAs in the soleus muscle were investigated using real-time reverse transcription-polymerase chain reaction (real-time RT-PCR).

## Methods

### Animal surgery and tissue preparation

The experiments were performed on adult male Sprague-Dawley rats weighing 250-300 g. The experimental rats were randomly grouped into sham control, denervation, entrapment, or decompression, with 6 rats for each indicated evaluation time point. The rats were anesthetized using an intraperitoneal injection of 400 mg/kg chloral hydrate. The sciatic nerves were exposed at the mid-thigh level after a dorsolateral skin incision had been made and the fascia had been split between the gluteus and biceps femoris muscles. The right sciatic nerve was gently dissected from the surrounding connective tissues from the gluteus muscle to the trifurcation of the sciatic nerve. In the denervation group, the sciatic nerve was severed at 1 cm proximal to the nerve trifurcation, which innervates all lower leg muscles including the soleus muscle, and we surgically removed a 1-cm segment of the proximal nerve. The proximal stump of the proximal nerve ending was ligated and transferred cephalically into the proximal thigh muscle to prevent regenerative terminal or spontaneous collateral sprouting. In the entrapment group, a 1-cm piece of non-constrictive silastic tube (Baxter Healthcare, Deerfield, IL, USA) with an inner diameter of 1.3 mm and an outer diameter of 2.0 mm was placed around the segment of the sciatic nerve just distal to the gluteus muscle, as previously reported [25]. In the group of rats that underwent surgical decompression at 6 months after the placement of the silastic tube, atraumatic removal of the silastic tube was performed under operative microscopy using the same operative and anesthesia techniques described above [25]. Using this entrapment/decompression model, we previously demonstrated the myopathy in a histopathological picture during nerve entrapment and observed some recovery of myopathy after surgical decompression [26]. The rats were sacrificed postoperatively at the indicated time points to collect the L4-L6 spinal hemi-segment, ipsilateral dorsal root ganglia (DRGs), and soleus muscle of the experimental limb. All operations were performed under an operative microscope by the same surgeon. The specimens were placed in isopentane, frozen in liquid nitrogen, and stored at -80°C. All housing conditions, surgical procedures, analgesia, and assessments were performed according to the Animal Care Guidelines and protocols approved by the Animal Care Committee at Chang Gung Memorial Hospital.

### RNA isolation

Total RNA was extracted using the mirVana miRNA Isolation Kit (Ambion, Austin, TX, USA). The purified RNA was quantified by determining the absorbance at 260 nm using an SSP-3000 Nanodrop spectrophotometer (Infinigen Biotechnology Inc., City of Industry, CA, USA). For the miRNA array and whole genome expression analyses, the quality of the purified RNA was assessed using a Bioanalyzer 2100 (Agilent Technology, Santa Clara, CA, USA). Total RNA (2 μg) was reverse transcribed into cDNA in a total volume of 20 μL using the High Capacity cDNA Reverse Transcription Kit (Applied Biosystems, Foster City, CA, USA). Each miRNA cDNA (10 ng) was obtained using the TaqMan miRNA Reverse Transcription Kit (Applied Biosystems) with the miRNA being reverse transcribed from the target miRNA using sequence-specific stem-loop primers.

### Expression of miRNAs

The experimental spinal segments, DRGs, and muscles from 3 rats in each group (sham control, denervation, entrapment, and decompression) were used for the microarray analyses. In brief, 100 ng of total RNA was dephosphorylated using 11.2 units of calf intestine alkaline phosphatase (GE Healthcare Life Sciences, Uppsala, Sweden) for 30 min at 37°C. The reaction was terminated by heating the samples at 100°C for 5 min and immediate cooling to 0°C. Dimethyl sulfoxide (DMSO, 5 μL) was then added; the solution was heated to 100°C for 5 min and immediately cooled to 0°C. Ligase buffer and bovine serum albumin (BSA) were added and ligation was performed by adding pCp-Cy3 (50 μM) and 15 units T4 RNA ligase in 28 μL. The samples were then incubated at 16°C for 2 h. The labeled miRNAs were desalted using MicroBioSpin6 columns (Bio-Rad, Hercules, CA, USA). Subsequently, 2× hybridization buffer was added to the labeled mixture to a final volume of 45 μL. The mixture was heated for 5 min at 100°C and immediately cooled to 0°C. Each 45-μl sample was hybridized to an Agilent Rat miRNA Array (G4473A) at 55°C for 20 h; this array includes 350 rat miRNAs based on the Sanger miRBase (release 10.1). After hybridization, the slides were washed at room temperature for 5 min in Gene Expression Wash Buffer 1 and then for 5 min in Gene Expression Wash Buffer 2. The slides were scanned on an Agilent microarray scanner G2565A at 100% and 5% sensitivity settings. Agilent Feature Extraction software (version 9.5.3) was used for image analysis, and normalization software was used to quantify the signal and background intensity for each feature. The substantially normalized data were analyzed using the rank-consistency filtering LOWESS method. The microarray data were analyzed using GeneSpring GX 7.3.1 software (Agilent Technologies). We selected those differentially expressed miRNA genes that showed a 2-fold change in their expression level between the experimental specimens and the sham control group. These differentially expressed genes were applied to hierarchical cluster analysis using average linkage and Pearson correlation as a measure of similarity. The miRNA array data have been deposited in the Gene Expression Omnibus (accession number [GEO: GSE22181])

### Quantification of miRNA expression

miRNA expression was quantified using real-time RT-PCR on the Applied Biosystems 7500 Real-Time PCR System (Applied Biosystems) to verify the upregulated miRNA targets detected by the miRNA array from the spinal segments (miR-384-3p, miR-325-5p, miR-342-5p, and miR-340-5p) and DRGs (miR-21) in the denervation and sham control groups, and the muscle-specific miRNAs (miR-1, miR-133a, and miR-206) in the soleus muscles of the sham control, entrapment, and decompression groups. The expression of each miRNA was represented relative to the expression of the small RNA 4.5 S internal control. We calculated the fold-expression of induction as the relative expression value obtained from 6 samples compared to that from the sham control group. The comparison between the groups included ANOVA and an appropriate post hoc test to compensate for multiple comparisons (SigmaStat; Jandel, San Rafael, CA, USA). P-values < 0.05 were considered significant.

### In situ hybridization

We used *in situ *hybridization to localize miR-21 expression in the DRG sections. The frozen section specimens were acquired from 10-μm thick transverse cross sections from the middle of each fresh specimen using a cryostat (Cryostat Leica CM3050, Bannockburn, IL, USA). Cryosections were fixed in 4% paraformaldehyde, acetylated in 0.1 M triethanolamine (pH 8.0), and then treated with proteinase K. After washing with phosphate-buffered saline (PBS), the specimens were incubated with 10 pmol of locked nucleic acid (LNA)-modified oligonucleotide probe (Exiqon, Woburn, MA, USA) complementary to *Rattus norvegicus *(rno)-miR-21 and labeled with digoxigenin (DIG) at 50°C overnight. For the sham control experiment, the tissue sections were hybridized with rno-U6 probes or without rno-miR-21 probes as positive or negative controls, respectively. After hybridization, the slides were washed in 50% formamide, 1× saline sodium citrate (SSC), and 0.1% Tween-20 at 50°C, followed by washes in 0.2× SCC and PBS at room temperature. The specimens were then incubated with the blocking solution followed by a horseradish peroxidase (HRP)-conjugated anti-DIG antibody. The signal was enhanced using the TSA Plus DNP HRP system (PerkinElmer Life Sciences, Boston, MA, USA). The hybridized probes were detected and visualized by a color reaction with 3,3'-diaminobenzidine (DAB).

## Results

### Expression profile of the miRNAs in the muscle

**Figure 1:**
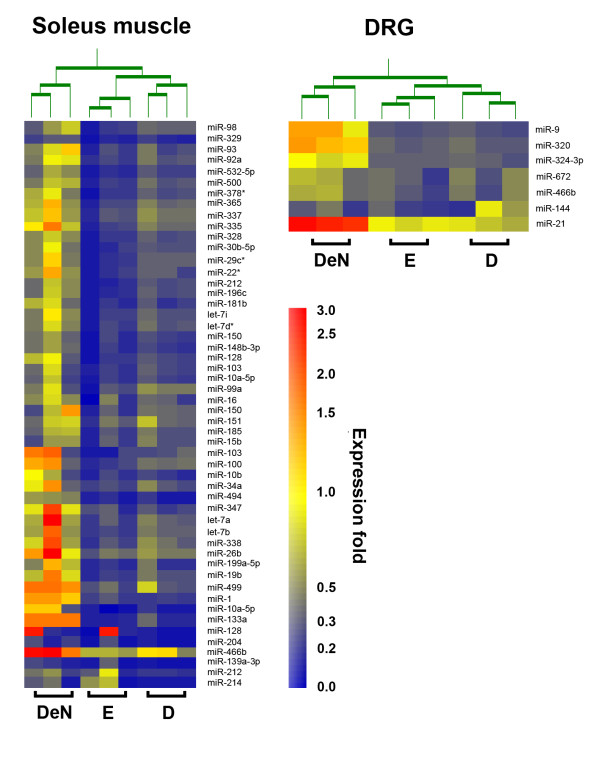
**Hierarchical cluster analysis**. Using a hierarchical method, a clustering graph was created from those miRNAs with increased (red) or decreased (blue) fold of expression from 3 soleus muscles (left graph) and dorsal root ganglia (right graph) in the group of rats that sustained denervation for 6 months, entrapment for 6 months, or decompression for 3 months against those from the sham control group.

miRNA array analysis indicated that approximately 13.4% (n = 47) and 11.4% (n = 40) of all rat miRNAs tested (350 unique miRNAs) were significantly decreased at 6 months of entrapment neuropathy and at 3 months after decompression, respectively, in the soleus muscle (Additional file 1: Table S1). Notably, there were 37 overlapping miRNAs under these 2 conditions and no miRNAs were upregulated in the soleus muscles of these 2 groups. Surgical decompression appeared to not remarkably alter the general expression of the downregulated miRNAs in the innervated muscle in entrapment neuropathy. In contrast, 3 miRNAs (miR-499, miR-1, miR-133a, and miR-466b) were upregulated in the denervated muscle and 3 miRNAs (miR-329, miR-204, and miR-139-3p) were downregulated after 6 months. A hierarchical cluster analysis of all significantly dysregulated miRNAs is shown in Figure 1, illustrative of miRNAs differentially expressed under these 3 conditions. We were able to separate the samples into denervation, entrapment, and decompression by performing unsupervised hierarchal clustering analysis.

### miRNA expression profiles in the neurons

**Figure 2:**
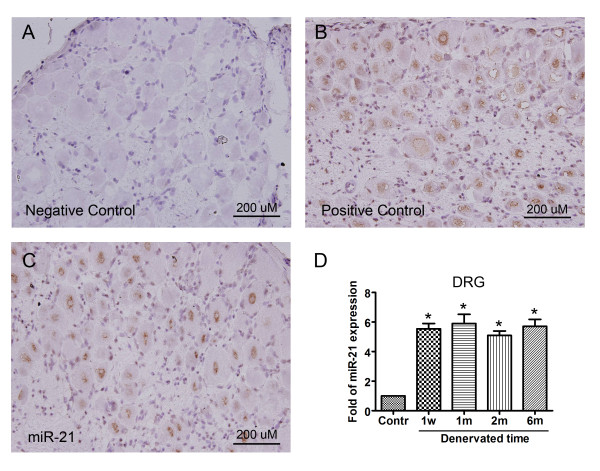
***In situ *hybridization analysis**. In comparison with the negative control (Figure 2A), *in situ *hybridization analysis revealed that the positive control probe rno-U6 was abundantly and diffusely expressed in the perinuclear region of the neurons in the denervated dorsal root ganglia (DRGs) (Figure 2B). Intense signals for miR-21 in the perinuclear region of the neurons were also observed in the tissue sections at 1 month after denervation injury by using digoxigenin-labeled miR-21 probes (Figure 2C). Real time RT-PCR revealed that the expression of miR-21 in the DRGs was increased by ~6 fold; it was detected 1 week after denervation and lasted for up to 6 months (Figure 2D). Bars represent means ± standard error of 6 independent experiments; *, P < 0.05 vs. sham control.

In the DRGs, 6 miRNAs (miR-9, miR-320, miR-324-3p, miR-672, miR-466b, and miR-144) were significantly downregulated in the entrapment group and 3 miRNAs (miR-9, miR-320, and miR-324-3p) were significantly downregulated in the decompression group. Although there also appeared to be a decrease of miR-672, miR-466b, and miR-144 in the decompression group, it was not statistically significant in all 3 specimens. No miRNAs were upregulated in the DRGs of the entrapment and decompression groups. On the other hand, after sciatic nerve denervation, we observed 1 downregulated miRNA (miR-144) and 1 upregulated miRNA (miR-21) in the DRGs (Figure 1). To localize the expression of miR-21 in the DRGs, *in situ *hybridization of miR-21 was performed to differentiate whether the upregulation occurred in the neurons or the interstitial connective tissue. As shown in Figure 2, in comparison with the negative control (Figure 2A), the positive control probe rno-U6 was abundantly and diffusely expressed in the perinuclear region of the DRG neurons after 6 months of denervation (Figure 2B). In addition, using DIG-labeled miR-21 probes, intense signals for miR-21 were also observed in the perinuclear region of the neurons (Figure 2C). Real time RT-PCR revealed that the expression of miR-21 in the DRGs was detected after 1 week of denervation with an ~6-fold increase that lasted for up to 6 months (Figure 2D). We were able to separate the samples into denervation, entrapment, and decompression by performing unsupervised hierarchal clustering analysis.

In the L4-L6 spinal segments, no dysregulated miRNAs were detected using the miRNA array in the entrapment and decompression groups. In the denervation group, 4 miRNAs (miR-384-3p, miR-325-5p, miR-342-5p, and miR-340-5p) were significantly upregulated, but no miRNA was downregulated in the spinal segment specimens after 6 months. However, using quantitative real-time RT-PCR to independently measure the relative expression of these 4 selected miRNAs in the spinal segment samples, none of them could be validated and all miRNAs were upregulated.

### Expression of muscle-specific miRNAs in the entrapment and decompression groups

**Figure 3:**
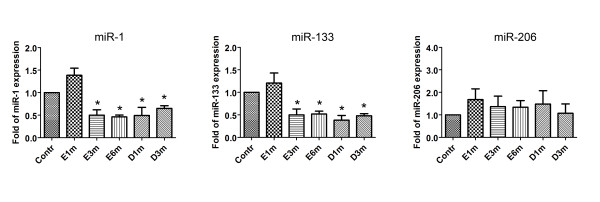
**Expression of muscle-specific miRNAs using quantitative real-time PCR**. After entrapment, the expression of miR-1 and miR-133 was significantly decreased to ~50% of those observed in the sham control group at 3 and 6 months after entrapment. After decompression, miR-1 and miR-133a levels were unchanged and sustained a significant decrease at 1 and 3 months later, respectively. There were no statistical differences in the expression of miR-206 at the indicated time points after nerve entrapment and after decompression. Bars represent means ± standard error of 6 independent experiments; *, P < 0.05 vs. sham control.

After nerve entrapment using a silastic tube, we observed the downregulation of miR-1 and miR-133a in the soleus muscle at 3 months after its insertion that lasted until at least the 6-months time point (Figure 3). Real-time RT-PCR analysis revealed an ~50% decrease in the expression levels of miR-1 and miR-133a at 3 and 6 months after entrapment, whereas the levels of miR-1 and miR-133a were unchanged and then decreased after decompression for 1 and 3 months, respectively. The expression patterns of miR-1 and miR-133a were similar after entrapment and decompression. In contrast, there were no statistical differences in the expression of miR-206 after entrapment and decompression.

## Discussion

According to the degrees of nerve injury defined on the basis of the histopathology and pathophysiology of nerve injury [27], entrapment neuropathy would be graded as a grade II or III injury [28], but not as a grade V injury after nerve transection (neurotmesis). Electron microscopic analysis of axonal integrity in entrapment neuropathy has also shown no morphometric evidence of axonal injury [28,29]. Therefore, it is not surprising that neurons and innervated muscles exhibit different responses between entrapment neuropathy and denervation injury. In this study, we demonstrated that entrapment neuropathy resulted in miRNA expression patterns that differed from those in denervation injury in the DRGs and the innervated soleus muscle. Compared to the 3 upregulated and the 3 downregulated miRNAs in the denervated muscle, we observed more extensively downregulated miRNAs (13.4%, 47 of the 350 rat miRNAs) in entrapment neuropathy. In the DRGs, there was 1 downregulated and 1 upregulated miRNA after nerve denervation, but there were 6 significantly downregulated miRNAs after entrapment neuropathy. We were able to separate the DRGs or muscle samples into denervation or entrapment neuropathy by performing unsupervised hierarchal clustering analysis. Considering that a single miRNA may target multiple transcripts [30] or activate an extremely powerful mechanism to dynamically adjust the cell's protein content and influence cellular physiology [31,32], it is reasonable to assume that, despite the fact that there are still unknown downstream target genes and functions, the epigenetic regulation of neurons and innervated muscle is different in response to entrapment neuropathy and denervation injury.

Moreover, the expression of muscle-specific miRNAs was different between entrapment neuropathy and denervation injury. Previously, in a rat model of sciatic nerve denervation, in the absence or presence of nerve microanastomosis [22], we demonstrated that the expression patterns of miR-1 and miR-133a were similar in the soleus muscle after denervation and reinnervation. The expression of miR-1 and miR-133a increased in the muscle after 4 months of denervation and reinnervation. On the other hand, the expression of miR-206 was only significantly increased at 1 month after reinnervation, but not after denervation, and lasted for at least 4 months. In this study, there was an ~50% decrease in the expression levels of miR-1 and miR-133a at 3 and 6 months after entrapment as well as after 1 and 3 months of decompression. In contrast, we found no statistical difference in the expression of miR-206 during nerve entrapment or after decompression. miR-1 and miR-133a are transcribed from a common pre-miRNA precursor in the miR-1/miR-133a locus that generates different primary transcripts [33]. Thus, it is not surprising to discover that the expression patterns of miR-1 and miR-133a were similar. In contrast, the expression pattern of miR-206 was found to be independent from those of miR-1 and miR-133a. However, the expression response of these 3 muscle-specific miRNAs to entrapment neuropathy and denervation injury was different. It has been reported that the expression of miR-1 and miR-133a decreased during skeletal muscle hypertrophy after 7 days of functional overload in rats. The decreased expression of miR-1 and miR-133a was suggested to compensate for the overload by removing the posttranscriptional repression of the necessary target genes [34]. In addition, miR-206 is required for the efficient regeneration of neuromuscular synapses after acute nerve injury [35]. At 3 weeks after surgical denervation, the reinnervation of the denervated muscles by motor axons was delayed in miR-206^-/- ^mice, but wild-type and miR-206^-/- ^mice exhibited similar degrees of muscle atrophy [35]. However, the role of the muscle-specific miRNAs to entrapment neuropathy or denervation injury remains to be investigated.

We also demonstrated that, after decompression, the miRNA expression patterns in the soleus muscles were similar to those in entrapment neuropathy. Although we were able to separate the muscle or DRGs samples into entrapment or decompression by performing unsupervised hierarchal clustering analysis, 37 of the 47 miRNAs downregulated in entrapment neuropathy were among the 40 miRNAs downregulated after decompression; besides, the expression profile of muscle-specific miRNAs was not different after entrapment neuropathy or decompression. Surgical decompression appeared to not remarkably alter the general expression of the downregulated miRNAs in the muscle during entrapment neuropathy, leading us speculate that is it possible that the unaltered posttranscriptional regulation attributes to some refractory response of the muscle after surgical decompression. The identification of the target genes of the differentially expressed miRNAs using a combined approach of an miRNA prediction algorithm and whole genome expression analysis under the condition of gain-of-function or loss-of function of each miRNA would be helpful to elucidate the role of each miRNA. However, considering the lack of transgenic or knock-out rodents and the expensive nature of antisense oligonucleotides or miRNA inhibitors, this goal could not be easily achieved and requires further extensive investigation.

In this study, we had demonstrated an ~6-fold increase in the expression of miR-21 in DRG neurons at 1 week after denervation that lasted for up to 6 months. miR-21 is one of the most prominent miRNAs implicated in human malignancies [36]. It is highly expressed in glioblastomas and functions as an oncogenic and anti-apoptotic factor, while it is expressed at basal levels in other brain tumors and in the normal brain [37,38]. miR-21 was reported to play a cytoprotective role against injury via its target genes [39,40]; however, the role and target genes of miR-21 expression in the DRGs after denervation were beyond the scope of this study. Besides, we did not detect dysregulated miRNAs in the L4-L6 spinal segments of the entrapment and decompression groups using miRNA arrays. Although 4 miRNAs were significantly upregulated in the miRNA array experiment, their increased expression could not be validated using quantitative real-time RT-PCR. It should be noted that false positive and false negative targets are expected when using an miRNA array. In a comparison of six microarray platforms and one next-generation sequencing machine to detect the differential expression of miRNAs, Agilent, Ambion, and Exiqon microarrays had the highest rates of true differentially expressed calls with true positive/true negative rates of: Agilent, 0.90/0.86; Ambion, 0.91/0.91; and Exiqon, 0.82/0.85 [41]. Generally, quantitative real-time RT-PCR results are consistent with the results of the high-throughput microarray method in normal physiological or disease-related pathological conditions [42,43]; with few identified targets, microarray and next-generation sequencing data are regularly validated using quantitative real-time RT-PCR. Nevertheless, in a genomic miRNA analysis, microarrays are still the best choice for a standardized genome-wide assay that is amenable to high-throughput applications [41]. In addition, we speculated that the complex composition of the spinal segment tissue, which contains different neuronal and glial cells and different cells in the supporting connective tissue, may be the principal obstacle for a correct tissue dissection and subsequent quantification of miRNA expression. For these reasons, the interpretation of data acquired from microarray platforms needs to be carefully performed in specimens with complex components. In addition, further experiments on the tissues or neurons acquired with a more accurate method would be helpful to identify the miRNA expression profiles in the spinal segments.

## Conclusions

In the past few years, it has become clear that miRNAs are a fundamental part of coordinated gene regulation in different aspects of cell biology. This study has provided new insights into the role of miRNAs after sciatic nerve denervation, entrapment neuropathy, and decompression by demonstrating the differential regulation of miRNAs in the innervated neurons and muscles. However, the field of miRNA research is still in its infancy and requires further exploration to understand the role of miRNAs in these conditions. Further identification of their target genes and their function would help to elucidate the role of each miRNA involved.

## Competing interests

The authors have no personal financial or institutional interest in any of the drugs, materials, or devices described in this article.

## Authors' contributions

CHH and CSR were responsible for the design and coordination of the data acquisition and analysis, search for target genes via the computational algorithm, interpretation of the data, and the writing of the manuscript. YCC participated in the real-time RT-PCR experiments. JCJ, SFJ and PCL participated by providing and coordinating the resources. THL contributed to the animal surgery and acquisition of the study specimens. CJW and CJL were involved in the acquisition of the miRNA array and whole genome expression data. All authors read and approved the final manuscript.

## Pre-publication history

The pre-publication history for this paper can be accessed here:

http://www.biomedcentral.com/1471-2474/11/181/prepub

## Supplementary Material

Additional file 1 **Table 1**. Dysregulated miRNAs in the soleus muscle of rats in the denervation injury, entrapment neuropathy, and surgical decompression groups. † indicates the same miRNA target in the entrapment and decompression groups. Click here for file
