# Supplementary material for: Entrapment neuropathy results in different microRNA expression patterns from denervation injury in rats
Source: BMC Musculoskelet Disord. 2010 Aug 12;11:181. doi: 10.1186/1471-2474-11-181 (PMC2927509; doi:10.1186/1471-2474-11-181)
Supplement: Additional file 1 — Table 1. Dysregulated miRNAs in the soleus muscle of rats in the denervation injury, entrapment neuropathy, and surgical decompression groups. † indicates the same miRNA target in the entrapment and decompression groups. [file 1471-2474-11-181-S1.DOC]

| Denervation | | Entrapment | Decompression |
| --- | --- | --- | --- |
| up-regulated miRNAs | down-regulated miRNAs | down-regulated miRNAs | down-regulated miRNAs |
| miR-499 | miR-329 | †miR-98 | †miR-98 |
| miR-1 | miR-204 | †miR-329 | †miR-329 |
| miR-133a | miR-139-3p | †miR-93 | †miR-93 |
| miR-466b |  | †miR-92a | †miR-92a |
|  |  | †miR-532-5p | †miR-532-5p |
|  |  | †miR-500 | †miR-500 |
|  |  | †miR-378* | †miR-378* |
|  |  | †miR-365 | †miR-365 |
|  |  | miR-337 | †miR-335 |
|  |  | †miR-335 | †miR-328 |
|  |  | †miR-328 | †miR-30b-5p |
|  |  | †miR-30b-5p | †miR-29c* |
|  |  | †miR-29c* | †miR-22* |
|  |  | †miR-22* | †miR-212 |
|  |  | †miR-212 | †miR-196c |
|  |  | †miR-196c | †miR-181b |
|  |  | †miR-181b | †let-7i |
|  |  | †let-7i | †let-7d* |
|  |  | †let-7d* | †miR-150 |
|  |  | †miR-150 | †miR-148b-3p |
|  |  | †miR-148b-3p | †miR-128 |
|  |  | †miR-128 | †miR-103 |
|  |  | †miR-103 | †miR-10a-5p |
|  |  | †miR-10a-5p | †miR-16 |
|  |  | miR-99a | †miR-150 |
|  |  | †miR-16 | †miR-185 |
|  |  | †miR-150 | †miR-15b |
|  |  | miR-151 | †miR-103 |
|  |  | †miR-185 | †miR-100 |
|  |  | †miR-15b | †miR-10b |
|  |  | †miR-103 | †miR-494 |
|  |  | †miR-100 | †miR-347 |
|  |  | †miR-10b | †miR-1 |
|  |  | miR-34a | †miR-10a-5p |
|  |  | †miR-494 | †miR-133a |
|  |  | †miR-347 | miR-128 |
|  |  | let-7a | †miR-204 |
|  |  | let-7b | †miR-139-3p |
|  |  | miR-338 | miR-212 |
|  |  | miR-26b | miR-214 |
|  |  | miR-199a-5p |  |
|  |  | miR-19b |  |
|  |  | †miR-1 |  |
|  |  | †miR-10a-5p |  |
|  |  | †miR-133a |  |
|  |  | †miR-204 |  |
|  |  | †miR-139-3p |  |
